# Identifying Neighborhoods of Coordinated Gene Expression and Metabolite Profiles

**DOI:** 10.1371/journal.pone.0031345

**Published:** 2012-02-15

**Authors:** Timothy Hancock, Nicolas Wicker, Ichigaku Takigawa, Hiroshi Mamitsuka

**Affiliations:** 1 Bioinformatics Center, Kyoto University, Kyoto, Japan; 2 Laboratoire de Bioinformatique et Génomique Intégratives, Institut de Génétique et de Biologie Moléculaire et Cellulaire, Université de Strasbourg, Strasbourg, France; Kyushu Institute of Technology, Japan

## Abstract

In this paper we investigate how metabolic network structure affects any coordination between transcript and metabolite profiles. To achieve this goal we conduct two complementary analyses focused on the metabolic response to stress. First, we investigate the general size of any relationship between metabolic network gene expression and metabolite profiles. We find that strongly correlated transcript-metabolite profiles are sustained over surprisingly long network distances away from any target metabolite. Secondly, we employ a novel pathway mining method to investigate the structure of this transcript-metabolite relationship. The objective of this method is to identify a minimum set of metabolites which are the target of significantly correlated gene expression pathways. The results reveal that in general, a global regulation signature targeting a small number of metabolites is responsible for a large scale metabolic response. However, our method also reveals pathway specific effects that can degrade this global regulation signature and complicates the observed coordination between transcript-metabolite profiles.

## Introduction

The dynamics of metabolic networks are the product of complex interactions between genes, proteins and enzymes and metabolites. Since the introduction of DNA microarray technology, the expression signatures of metabolic networks have been extensively analyzed. An underlying assumption of these studies is that fluctuations in gene expression levels are mirrored in the protein and metabolite signals. Although it stands to reason that some relationship exists between metabolic gene expression and other observed metabolic responses, the inherent complexity of metabolism makes the validity of this assumption difficult to assess. Furthermore, it has previously been well established that the correlations between simple gene expression and protein or metabolic flux measurements are unreliable [1], [2]. Recently, many researchers have sought to elucidate these relationships through combined metabolomic and transcriptomic analyses. These combined analyses use techniques such as Gas Chromatography Mass Spectrometry (GC-MS) and microarrays to simultaneously measure changes in metabolite concentrations and gene expression [3], [4]. The integration of these two data sources provides the opportunity to more thoroughly understand how changes in gene expression are converted into metabolic responses.

The results of these combined studies have revealed that transcript and metabolite interaction is often quite complex. Intermediate steps between transcription and metabolite production such as post-translational modification [5], regulation or buffering expression by metabolite levels [6] have been found to seriously affect any simple relationship. However, studies have shown simple coordination between metabolite and expression exists although it is either locally restricted [7], around specific reporter reactions [8], or highly specific to environmental stress conditions [4], [9].

It is clear that intermediate steps such as post-translational modification and buffering have a pronounced effect on the transcriptome-metabolome relationship. However, the extent to which the network structure of metabolism impacts this relationship is unclear. It is known that metabolic gene expression is highly coordinated along pathways [10], [11] and that this coordinated structure is significantly rewired in response to an external stress. Clearly this regulated coordination of gene expression along metabolic pathways is intended to effect the protein and finally metabolite profiles. In this paper we investigate how the network structure effects the correlation between gene expression and metabolite profiles. To address this question we develop models to uncover the gene pathways with the most coordinated expression profiles and then use the expression profiles along these pathways to identify the potential target metabolites.

There are two main theories regarding how metabolic networks function and respond to external stimulus; robustness and modularity. Robustness can be observed as metabolic networks are surprisingly resistant to genetic [12] or metabolomic [13] perturbations. Modularity attempts to explain this observed robustness through densely connected community structure centering around critical genes and metabolites. This community structure provides backup pathways that are activated in response to an induced perturbation [14]. These backup pathways possess a branched structure connecting the densely connected subgraphs or modules [10], [15]. Such a branched structured graph connecting dense modules is the basis for the observation of modularity within metabolic networks [15], [16]. Metabolic network modularity implies that global regulation exists which activates specific modules of genes to produce a required metabolic process. Modularity places important metabolites in the center of these branched clusters [13], [17] and then assumes that within these modules a high level of coordinated gene expression exists surrounding these important metabolites. Therefore, the task of identifying these modules is synonymous to identifying the important metabolites which are required to reproduce specific metabolic processes.

Identifying the important metabolite which are driving the function of metabolic networks therefore also gives insights into the modularity and robustness properties of metabolic networks. To achieve this goal, network structural analysis methods such as network expansion models seek to identify the input metabolites which if provided as input can be used to synthesize all network elements [18]. Network expansion identifies these input metabolites by defining the scope of a set of input (seed) metabolites. The scope of a set of seed compounds is defined to be the set metabolites which can be produced using only the seed compounds as input into the network. The network expansion model determines which metabolites are included in the scope by imposing the known stoichiometric rules of the network. The imposition of stoichiometric rules on the scope definition means that a compound can only be added to the scope if all required substrates have already been included within the scope. The network expansion scope is found by a greedy search through the metabolic network which spans out from the seed compounds iteratively adds the newly produced compounds that satisfy the required stoichiometric constraints.

The concept of metabolite reachability through the pathways of a metabolic network is fundamental to the network expansion model. The network expansion model defines metabolite reachability as those compounds which can be reached from the seed compounds without violating the stoichiometric constraints of the network. However, this assumption ignores the regulatory dynamics present within underlying gene expression. Another approach to define the set of reachable metabolites is to consider the correlation between the expression of neighboring genes within the network. The modeling of correlated neighboring expression defines metabolite reachability as those compounds which can be reached by a pathway of connected genes with highly coordinated expression profiles. Shifting the definition of metabolite reachability from pathways of stoichiometry consistency to pathways of coordinated gene expression leads to efficient algorithms for identifying the most probable paths between two metabolites [11].

This paper is separated into two complementary analyses focusing on the metabolomic and transcriptomic stress responses of the *Escherichia coli K-12 MG1655* metabolic network over four different stress conditions. Firstly, we investigate the overall structure of metabolic gene expression and metabolite profiles. This preliminary investigation shows that in general transcript-metabolite correlation is sustained over a surprisingly long network distance away from any given target metabolite. This result highlights the requirement for a broader analysis involving longer pathways of coordinated expression rather than focusing on the immediately connected reactions of any given metabolite. Secondly we seek to further understanding of these results by proposing variant of the network expansion approach which seeks to identify the important metabolites which are driving the function of the network by extracting pathways of genes within significantly coordinated expression profiles.

In the past, extracting pathways of maximum coordinated expression has been known to be biased towards shorter path lengths [11], [19]. In this paper we overcome this path length bias through the use of a significance test to determine if a path between any two metabolites is non-random. We then use this pathway significance test to define the sustainable scope of each metabolite to be the list of all metabolites which can be reached by a significantly correlated expression path through the network. From this list of extracted metabolite scopes we identify the important metabolites within the network through an integer programing solution to the minimum set cover problem. The result is minimum list of metabolite scopes required to completely encompass all significantly correlated paths within the network. Additional information obtained in the minimum set analysis are hub genes which mark local centers coordinated expression that are used by many paths in metabolite scopes.

Finally we compare the profiles of these hub genes to the metabolite profiles within the minimum set. This comparison confirms our initial hypothesis that broad coordination between transcript and metabolite profiles exists and is sustained over long network distances. More specifically, our overall result shows that global regulatory stress responses are focused on controlling the profiles of a small number of critical metabolites that are dictating the entire network response. However, for more complex stress conditions we observe a reduction in the strength of the global regulatory signal in favor of a pathway specific regulatory response.

## Results and Discussion

### Metabolite and Network Distance Relationship

**Figure 1:**
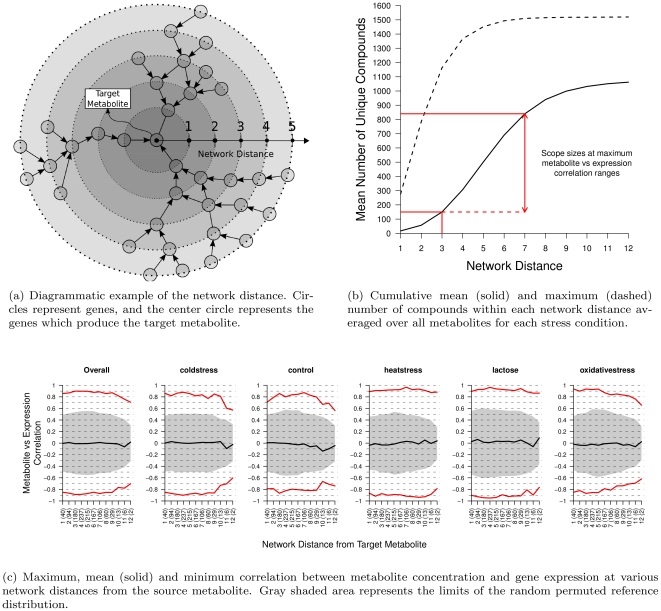
Overall view of the metabolite concentration and gene expression correlation at increasing network distances. In Figure 1c the numbers in brackets on the horizontal axis indicate the average number of unique genes at each network distance.

We first perform a preliminary investigation to identify the general network structure of the correlation between metabolite concentration and gene expression. Within each stress condition, for every metabolite which we have mapped to the network data, we treat this metabolite as the target metabolite. We then correlate the target metabolite concentration with the expression of all unique genes that have a direct path to produce the target metabolite at increasing network distances. This procedure is shown diagrammatically in Figure 1a. As many genes occur in many positions within the metabolic network we only consider the first instance of each gene, and remove any future references of that gene at longer network distances. Additionally to test if the resulting correlations are significant we compare against a reference distribution of correlation coefficients computed on 1000 random permutations of the metabolite concentrations with non-permuted gene expression values.

In Figure 1c we present the maximum, mean and minimum correlations observed between metabolite concentration and gene expression at each network distance. The shaded area in Figure 1c represents the range (minimum and maximum correlation coefficients) of the random permuted reference distribution. It is clear that the maximum and minimum correlations computed on the real data cannot be by chance as they lie well outside of the reference distribution. The results for each metabolite and stress condition are presented separately in the supporting Figure S1. The overall plot on the left side of Figure 1c is the additional average over each stress condition. An obvious feature of Figure 1c is that the maximum and minimum correlations are sustained over a longer of network distance of 3 to 7 reactions. The correlation values observed at these distances is relatively strong ranging between 0.7 to 0.9. Additionally, this result suggests that there may be strong path specificity in positively correlated transcript and metabolite concentration profiles as the mean correlation is approximately 0 and the minimum correlation mirrors the maximum correlation but with a negative sign.

The strong correlations observed in Figure 1c are similar strength to those found by [4] and [9]. Additionally our result also explains why inconsistent correlations between transcript and metabolite concentration were obtained when only immediately connected reactions are considered [7], or appear weak if the network structure is not taken into account [5]. In fact, such long distances are also indicative of a very general network response to external stress and suggest that hub or *reporter* reactions could be strongly controlling metabolite responses [8]. In Figure 1b we display the average number of metabolites that can be reached within each network distance and show that within a distance of 3 to 7 edges between 150 to 900 metabolites can be reached. This observation of long coordinated paths suggests that the effect of global regulation of a central module, rather than the immediately connected reaction to the target metabolite, is significantly affecting the specific metabolite profiles being produced. The activation of such a large sub-section of the metabolic network can be seen to be in agreement with the concept of modularity as it implies that many entire subnetworks show coordinated activation in response to stress. Additionally this result also implies that this global regulatory stress response is a large and non-specific effect which is activating multiple pathways to key metabolites, therefore reinforcing the robustness of the response.

### Minimum Set Extraction

The overall analysis to identify the minimum sets and analyze its structure for each stress condition has the following steps:

1. For each metabolite 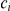, identify the set of all reachable compounds to define the scope, 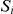 using our proposed path extraction algorithm in the Materials and Methods section.
2. Identify the minimum set of compound scopes 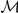, over all scopes 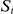, by solving the integer programming problem defined in the Materials and Methods section.
3. Collapse all paths included within the minimum set, 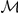, down into a single network of traversed reactions and weight each edge by the number of paths within which it is observed within 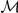. Then construct a maximum spanning tree of most commonly traversed reaction paths and extract the top 10 most connected hub reactions.
4. Correlate the gene expression of all hub reactions with the concentration profiles of the metabolites within the minimum set.

As this procedure contains many separate steps, at each stage we assess the validity of the current results. The validity of each pathway is assessed by its 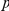-value within a distribution of all network pathways computed by Metropolis sampling [20]. However, extracting the scope of a single compound requires many 1000's of 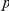-values to be computed. Therefore to minimize the false positive rate a Bonferroni correction was applied to a base significance threshold of 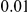 and corrects for the number of metabolites in the network.

Once all scopes have been extracted the size of each scope and length of all paths are compared to the expected scope sizes computed from our preliminary experiments in Figure S2. In the scope size distribution (supporting Figure S2) for each stress condition is shown to be highly positively skewed with larger scopes being more unlikely. The largest extracted scope size is found to be 954 for lactose, 950 for heat stress, 929 for cold stress and 843 for oxidative stress. These maximum scope sizes are consistent with our preliminary results on the transcript-metabolite network distance relationship which suggested scope sizes of approximately 850–900. The path length histograms (supporting Figure S2) show the average path length over all scopes and stress conditions is approximately 10 reactions and the distributions have a reasonably broad variance. This rather long average path length and broad variance confirms that extracting the most significant path is successfully removing the short path length bias known to exist in standard shortest path algorithms. Although the average path length is above the estimate of 7 to 8 correlated reactions (Figure 1b), it is not entirely unexpected given strongly coordinated expression profiles inherent in metabolic networks [10], [11].

Once all scopes for each stress condition have been extracted the minimum sets are computed. The results of the minimum set results are presented in Table 1. Table 1 reveals that the number of metabolite scopes included within the minimum sets is quite small, at most 27 scopes. A surprising result from Table 1 is that the number of compounds and reactions included are consistent over all stress conditions. Within each stress condition approximately 1000 compounds can be reached using approximately 1200 reactions, which equates to over half of the entire metabolic network.

**Table 1:** Minimum set cover solution summary statistics for each stress condition.

Stress Condition	Minimum Set Optimal Size	Number of Solutions	Combined Minimum Set Size	Number of Covered Compounds	Number of Covered Reactions
**coldstress**	25	4	27	1053	1217
**heatstress**	20	1	20	1022	1182
**lactose**	18	1	18	1053	1240
**oxidativestress**	22	6	24	1012	1159

Prompted by the large number of reactions included in the resulting minimum sets an additional validation was performed using the enrichment score from gene set enrichment analysis (GSEA) [21]. An enrichment test is performed to determine if the edges selected over all paths within the minimum sets are significantly positively correlated given all edges within the network. The results of this validation reveals each scope to comprise of a significantly correlated subnetwork with enrichment scores of 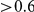 and the maximum enrichment scores over 1000 permuted gene sets to be 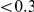. This result validates each minimum set to contain paths of significantly positively correlated edges and reveals that at over many stress conditions *Escherichia coli* is highly coordinating the expression of over 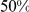 of its metabolic network.

However, it is expected that much of this coordination will be part of normal cell functioning and not in response to the external stress. The effect of the external stress will be to activate or deactivate specific pathways which will rewire the correlation structure of each network, but as Table 1 shows the rewiring is unlikely to alter the number of reachable compounds or the number of reactions used. This finding agrees with the observations of [7] which show that changes in transcription or enzyme abundance can change individual reaction rates but overall do not affect the homeostasis of the global metabolic network. As a result we seek to analyze the structure of each re-wired network by identifying hub genes within frequently traversed paths through the network and correlate these hub genes with the metabolite profiles within the minimum set.

### Minimum Set Analysis

**Figure 2:**
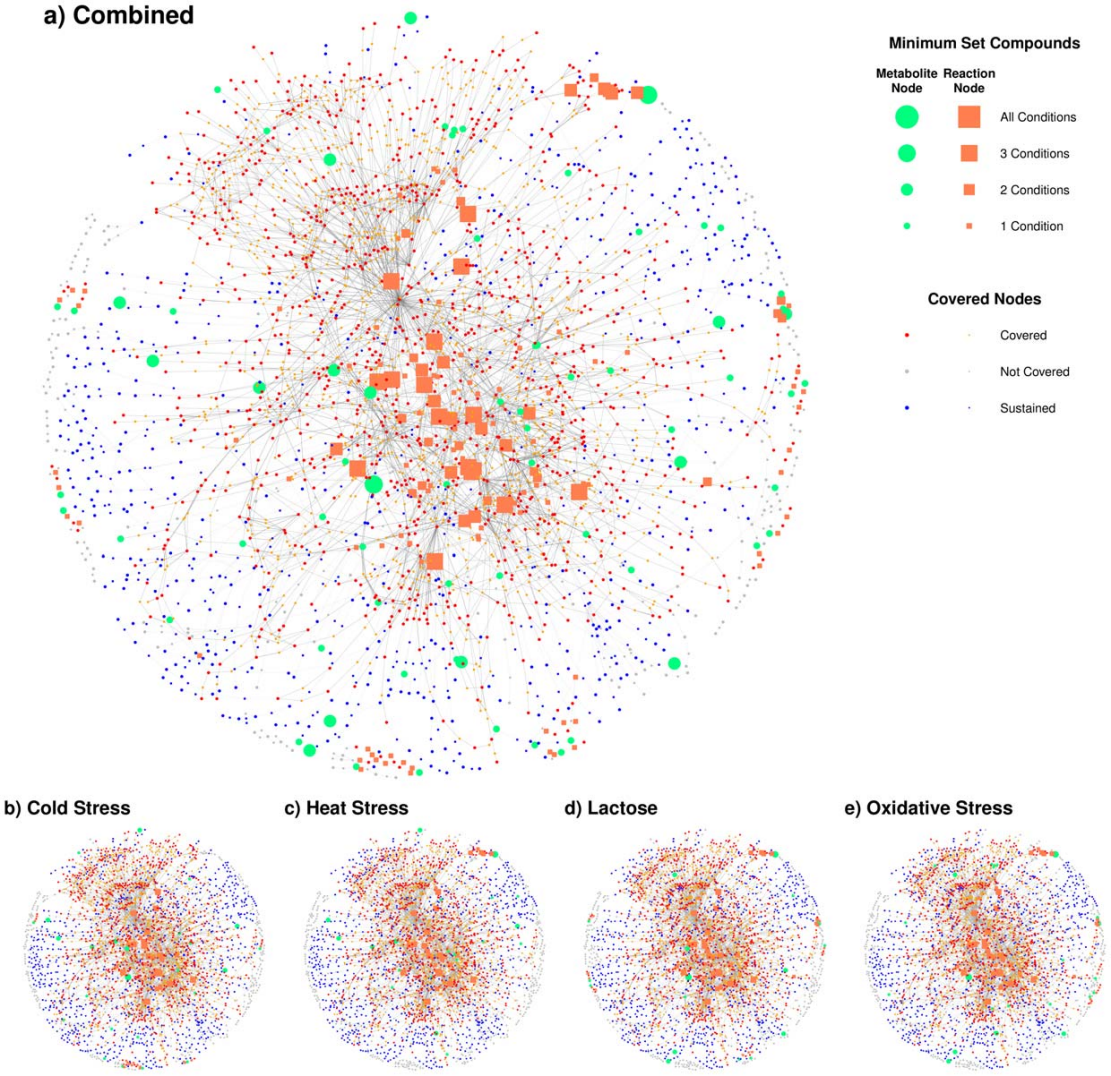
Minimum set network visualization for all stress conditions combined, and for each separately. Green circles are minimum set metabolites and orange squares are hub reactions. The node size reflects how many stress conditions each node is observed. Small red circles and orange squares are metabolites which can be produced by a significant path and reactions respectively. Blue nodes are metabolites which are included in a path included within the minimum set, but have no significant path terminating at that metabolites. Gray nodes are not included in any path.

In Figure 2 we visualize the structure of each minimum set, and the combined view over all minimum sets. In Figure 2 green circles are minimum set metabolites and orange squares are hub reactions. Small red circles are metabolites which are produced by a significant path ending at that metabolite. Blue nodes are metabolites which are included in a path within the minimum set, but have no significant path ending at that compound. Gray nodes are not included in any path.

The relative sizes of the nodes in Figure 2 indicate the number of stress conditions whose minimum sets include that node. The stress conditions of each network are heavily overlapping and share many common reaction hubs which form a large central sub-graph within the metabolic network. Although this large amount of overlap is expected as each minimum set includes over half compounds and reactions of the entire network (Table 1) the sheer size of the central cluster and the number of common hubs illustrates the strong interconnectivity of metabolism. This interconnectivity reinforces the observation that metabolic networks are very robust as the density of these bipartite graphs is proportional to the number of paths which connect the critical metabolites.

A more detailed and annotated view of the minimum sets in form of a bipartite graph is available in the supporting Figure S3. The bipartite graphs are constructed using the minimum set metabolites as the first node set and the common hub reactions included within each minimum set scope as the second node set.

The majority of common reaction hubs across each stress condition, aspartate transaminase, glutamine synthetase, pyruvate formate lyase, glutamate dehydrogenase (NADP), glutamine-fructose-6-phosphate transaminase, acetylornithine transaminase, phosphoenolpyruvate synthase, nitrate reductase, cysteine synthase, succinate:fumarate antiporter, methionine adenosyltransferase, are directly connected to the centrally located amino acid metabolism pathway of alanine, aspartate, asparagine, glutamate metabolism [22]. The metabolism of alanine, aspartate, asparagine, glutamate can be regulated in a large part by rpoS (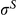) [23]–[25] and H-NS [26], [27] which are known to be general stress response factors of *Escherichia coli*. The central location and strong stress responsive regulation of alanine, aspartate, asparagine, glutamate metabolism suggests that this pathway is a hub pathway for critical source metabolites required for *Escherichia coli* stress response. Therefore, the regulation profiles of the genes along this pathway strongly determine the response profiles of the downstream connected pathways. Furthermore, this result suggests that tight regulation of a few centrally located metabolites can be sufficient to create an entire network stress response.

**Figure 3:**
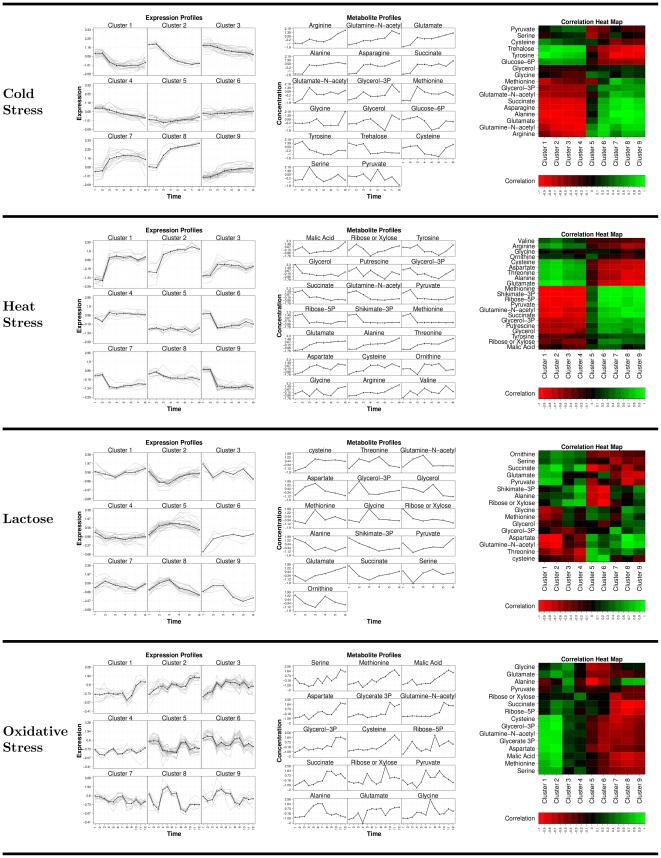
(**left column**) Clustered gene expression profiles (z-scaled) for each hub reaction; (**center column**) Metabolite profiles (z-scaled) for all metabolites in the minimum sets; (**right column**) A correlation heat map correlating the mean hub expression cluster profiles with each metabolite profile, green indicates strong positive correlation and red indicates strong negative correlation.

To validate our assertion that expression regulation of the alanine, aspartate, asparagine, glutamate metabolism are mirrored in the metabolite profiles we compare their profiles in Figure 3. In Figure 3 in the left most column, for each stress condition, we cluster the gene expression profiles of the identified hub reactions using 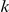-means set to 9 clusters. The central column in Figure 3 shows the metabolite profiles over time of the minimum set metabolites or hub reaction enzyme targets. The profiles of each metabolite are then correlated with the mean expression profile of each cluster and presented in the correlation heat map in the right column of Figure 3. Figure 3 shows that cold and heat stress have a very clear gene expression signature which is consistently correlated with the metabolite profiles. However, for lactose and oxidative stress the gene expression profiles are more complex and the correlation signature with the metabolite profiles is less consistent. Additionally what is immediately obvious is that the expression profiles are generally smoother and clearer than the metabolite profiles. The pronounced correlations surrounding amino acid metabolism and the more specific metabolite response compared to the transcript responses are observations which agree with those of [4].

For cold and heat stress, the transcript response is a sudden increase or decrease in expression levels. This profile jump is mirrored by some metabolites such as alanine, glutamate, asparagine, and succinate in cold stress and glycerol, ribose-5P, shikimate, methionione in heat stress. However it is observed, particularly in heat stress, the metabolites may respond to the jump in transcript levels by a smooth increase or decrease in concentration. Interestingly the clearest transcript/metabolite profile agreements are generally found within the explicit amino acid biosynthesis minimum set compounds. However as the distance from the amino acid biosynthesis pathways increases the profiles become more divergent e.g. glycine, glycerol, cysteine, serine. This indicates that although in general amino acid transcript and metabolite profiles are tightly coupled in *Escherichia coli's* cold and heat stress response, as the distance from this central pathway increases, so does the number of metabolites profiles that deviate from the jump profile. However these deviations occur at a later time in the experiment indicating that they are either due to pathway specific regulation or to a network latency from the stress response centered on amino acid biosynthesis e.g. glycine, glycerol, glucose-6P, trehalose and cysteine in coldstress and glycine, valine, malic acid, tyrosine and ornithine in heatstress. This agrees with the observation that protein degradation is increased in response to stress [28] resulting in the increased availability of amino acids which are then used to produce new proteins required for stress adaptation [29].

The transcript-metabolite correlation strength clearly degrades for lactose and oxidative stress responses indicating a significantly more complex response for these conditions. The weakening correlation signal for these two stress conditions is likely due to the effect of the additional metal ion enzyme cofactors that are included with the minimum set metabolites for these stress conditions (supporting Figure S3). The requirement for specific enzyme cofactors in addition to central activation of alanine, aspartate, asparagine, glutamate metabolism by rpoS and H-NS in the response to lactose and oxidative stresses indicates that for these conditions *Escherichia coli* requires additional pathway specific regulation. The shift from glucose as a major carbon source to lactose requires the ability to metabolize 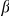-galactosidase which is known to require Zn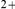 ions cofactors and is correlated with Cu, Mn, Ni and Co ions abundance [30]–[33]. Additionally iron and nickel are known to be cofactors in catalyzing the superoxide removal under oxidative stress [34] and in catalyzing reactions on the pentose phosphate pathway known for its detoxification response to oxidative stress [35], [36]. The effect of this more specific stress response is the drop in strength of the transcript-metabolite correlation signal. Although in Figure 3 strong correlations are still observed with central alanine, aspartate, asparagine, glutamate metabolites, more distant metabolites from this central pathway are effected by pathway specific regulatory responses and therefore show weaker transcript-metabolite correlations.

Overall we have shown that the concepts of metabolic network modularity and robustness can be reflected in the metabolite profiles. Furthermore strong correlations can exist between metabolite and gene expression, however the effect of the full metabolic network must be considered as these correlations occur at long network distances and are a result of global network regulation. This global regulation can be clearly seen in the strong transcript-metabolite correlation structure for cold and heat stress (Figure 3). However, for stress conditions such as a carbon source shift from glucose to lactose or oxidative stress the transcript-metabolite correlation profiles are significantly weaker. This is likely due to the effect of pathway specific regulation, such as post-transcriptional modification or the limitation of specific metabolites. This is hinted by the inclusion of metal ion cofactors within the cover sets of these conditions. These cofactors catalyze additional reactions which are specifically required to respond to these stress conditions. The effect of these enzymes is altering the metabolite concentrations through specific reaction kinetics and therefore cannot be reflected in the transcriptional response.

## Materials and Methods

### Network and Data Processing

The specific network reconstruction used is iAF1260 for Escherichia coli K-12 MG1655 [37] and was sourced from the BiGG database [38]. This network contains 1972 unique metabolic compound entries and 1944 reaction entries. Before any pathway analysis the network was preprocessed into a network of connected reactions. The preprocessing connects neighboring reactions by their substrate and product compound dependencies. Edge weights 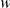 are then assigned between each connected reaction pair to be the maximum Pearson correlation between the expression profiles computed for all pairwise gene combinations from the gene sets of each connected reaction.

However constructing a network based solely upon substrate-compound dependencies has two consequences. Firstly it collapses the complex substrate-product compound dependencies into simple linear pathways where an edge is drawn between every substrate and compound pair of each reaction. This is simplification is a key difference between our approach and the original network expansion method [18] and is required to implement our approach of efficiently extracting pathways of significantly coordinated expression. This shifts the network expansion scope definition from the original set of feasible metabolites that can be produced from a collection of seed compounds and constrained by the network stoichiometry to the set of metabolites which are encompassed by the significantly coordinated expression signature which spans out from the set of seed compounds. Secondly the simplification creates huge numbers of redundant edges due to ubiquitous reaction cofactors and currency compounds, such as ATP, which are connected to most metabolites but do not create biologically interesting pathways. To solve the redundancy issue after conversion to the reaction network we remove all edges considering the following compounds, 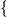 *H* 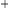 *, CoA, H* 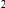 *O, CO* 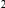 *, Orthophosphate, ATP, ADP, AMP, FAD, FADH2, GDP,GTP, NAD, NADH, NADP, NADPH, UTP* 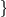. This list of compounds edges to be removed was generated to agree with the Pathfinder tool of Reactome [39], [40]. Finally at the end of this network pre-processing the resultant network has 9334 edges connecting neighboring reactions.

The dataset used is a combination dataset of time course gene expression and metabolite profiles for Escherichia coli K-12 MG1655 [4]. This study was performed on 4 stress and 1 control conditions; oxidative stress (**oxidativestress**), Glucose-Lactose Diauxic Shift (**lactose**), Heat Stress (**heatstress**), cold stress (**coldstress**) and the control condition (**control**). The expression dataset was downloaded from GEO (GSE20305) [41] and the raw metabolite dataset was sourced from the supplementary information website of the original paper. The metabolite dataset identifies time course data for 188 metabolites (95 could be positively identified, 58 chemically classified and 35 with an unknown structure). The location of specific metabolites stored in the raw data within metabolic network was done by a manual search matching the names of the metabolites found within the metabolite profiles data file with the SBML compound names found within the metabolic network. This search was able to identify 39 unambiguous metabolites that were contained within both the experimental data and the metabolic network. The microarray data was 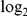 normalized and the metabolite data was normalized according to the instructions within the supplementary section of [4]. The experimental times for the microarray and metabolite data were aligned which results in 8 times for cold stress, control and heat stress, 6 times for lactose and 12 times for oxidative stress, where each time involves three biological replicates.

### Scope Extraction

We define a path beginning at compound **s** and terminating at compound **t** as an ordered sequence of reactions required to synthesize all required intermediate compounds and the final target compound **t**. Given a reaction network structure a path has the form specified in (1),
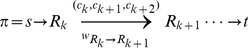
(1)where the entire path is denoted by 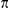, 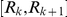 are path reactions, 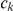 are the substrate and product compounds of 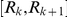 , 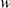 are the edge weights and **s** and **t** are pseudo-nodes added into the network to indicate the start and end vertices respectively of each path to be extracted. The edge weights, 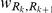, in (1) are the computed maximum correlation coefficients between the expression of all pair-wise combination of the genes within 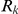, and 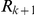.

We define the scope, 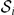, of a compound 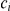 to be the list of all compounds which are connected to 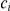 by a significantly highly correlated path. The scope extraction is done through a brute force search which simply enumerates through all pairs of specified start, **s**, and end, **t**, compounds. For example, given pair of compounds, 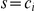 and 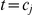; **s** is connected to all reactions where 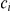 is a substrate and **t** is connected to all reactions where 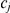 is a product. As many compounds occur in multiple positions within a metabolic network and have multiple substrate and product dependencies the pseudo-nodes **s** and **t** are usually reactions sets. For the case where **s** and **t** correspond to multiple reactions, to maintain computational efficiency we only include shortest significant path that spans between 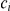 and 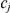 within the scope.

### Significant Path Ranking

To test if path is comprised of significantly coordinated expression profiles we first define the score of each path 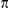 to be 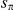 (2),
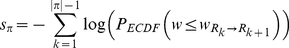
(2)where 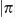 is the path length and 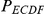 is the empirical cumulative distribution probability of an edge weight 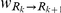 given all other edge weights within the network. If we then assume the edges along a given path are randomly and independently drawn from the network edge weight distribution, the 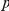-value of the path can be computed using (3) [11].
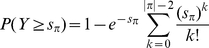
(3)Equation (3) simply computes the probability randomly and independently picking the path edge weights given all other weights in the network.

From [11], [19] we know that ranking only by (2) is biased towards shorter path lengths. However, ranking by 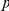-value corrects for the path length dependency by ensuring the extracted path is non-random. Our proposed approach is an extension on the standard shortest path algorithms where we use the observation that if we hold the path length (in terms of edges) constant the task finding the path of minimum 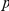-value is equivalent to maximizing the path score function (2). This suggests an algorithm which extracts the best path in terms of score between two vertices for all lengths, would also yield an algorithm to find the path of minimum 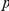-value. To extract a list of all best paths for all lengths between two vertices we use a dynamic programming algorithm. Once this list has been extracted we can readily find the most significant path over all lengths through direct evaluation of (3).

However our 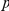-value computation assumes that each edge weight is independently drawn, which given the known network structure is unlikely to hold. To address these concerns we employ a Metropolis sampling algorithm [20]. The resulting Metropolis algorithm randomly samples candidate paths 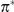 of all lengths 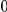 to 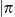 from the weighted network. The probability 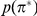, of each randomly sampled path 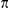 is stored and used as a reference distribution to compute the 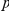-value of each shortest path identified by the algorithm. In the supporting methods (Methods S1) we show that computing the 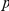-value from this reference distribution overcomes the randomly and independently drawn network edge assumption within (3). Further details of this sampling and pathway extraction methods are included in the supplementary methods.

### Metabolite Set Cover Analysis

Using our path ranking method we extract the scope of all compounds within the weighted metabolic network. This procedure will return, for each compound 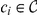 we define a scope 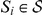 where 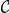 is the set of all compounds 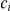, and 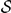 is the set of all compound scopes, 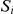. However set of all scopes in 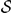 are likely to contain large amounts of overlap which correspond to highly coordinated sections of the metabolic network. The task now is to identify the minimum set of scopes 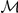 which can be used to represent this coordinated network structure. This task can be efficiently completed by solving the minimum set cover problem [42]. The result of this algorithm is a smaller set of compounds 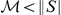 whose scope combined scopes encompasses all compounds that can be reached within the network. Therefore the combination of these compounds form a representative set of all significant paths within the network.

The minimum set cover problem seeks to find the minimum number of compound scopes 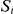 required to be selected such that each compound 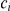 is included at least once over all scopes. This can be represented by the following binary integer programming problem,
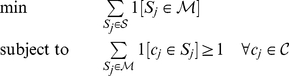
(4)where the minimum set of compounds is 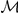 and 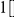 is a binary function which returns 1 when its bracketed logical operation is true. We solve this problem using the CPLEX software [43]. For any given collection of scopes, there is likely to be many solutions to (4). In this case we use the branch-and-cut method implemented in CPLEX to extract all possible solutions to (4). We then collapse all these solutions into one, non-optimal, but universal statement on the composition of the minimum set 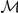.

## Supporting Information

Figure S1 Maximum, mean and minimum correlation between metabolite concentration and gene expression at various network distances from each source metabolite.
(PDF)

Figure S2 Scope size and path length distributions for each stress condition.
(TIFF)

Figure S3 Bipartite graph representing the structure of each stress conditions minimum set. Metabolite node sizes are proportional to the scope size of that metabolite. Hub reaction node size is proportional to how many times it is included in a minimum set metabolites scopes. In the case of long enzyme or metabolite names the first characters of the name are printed followed by the node label used within the SBML network file. The SBML file contains cellular compartment information for each metabolite {(c)ytosol, (e)xtraorganism, (p)eriplasm} which is printed after the ‘_’ in the metabolite name.
(PDF)

Methods S1 (PDF)
