# Supplementary material for: Identifying Neighborhoods of Coordinated Gene Expression and Metabolite Profiles
Source: PLoS One. 2012 Feb 15;7(2):e31345. doi: 10.1371/journal.pone.0031345 (PMC3280297; doi:10.1371/journal.pone.0031345)

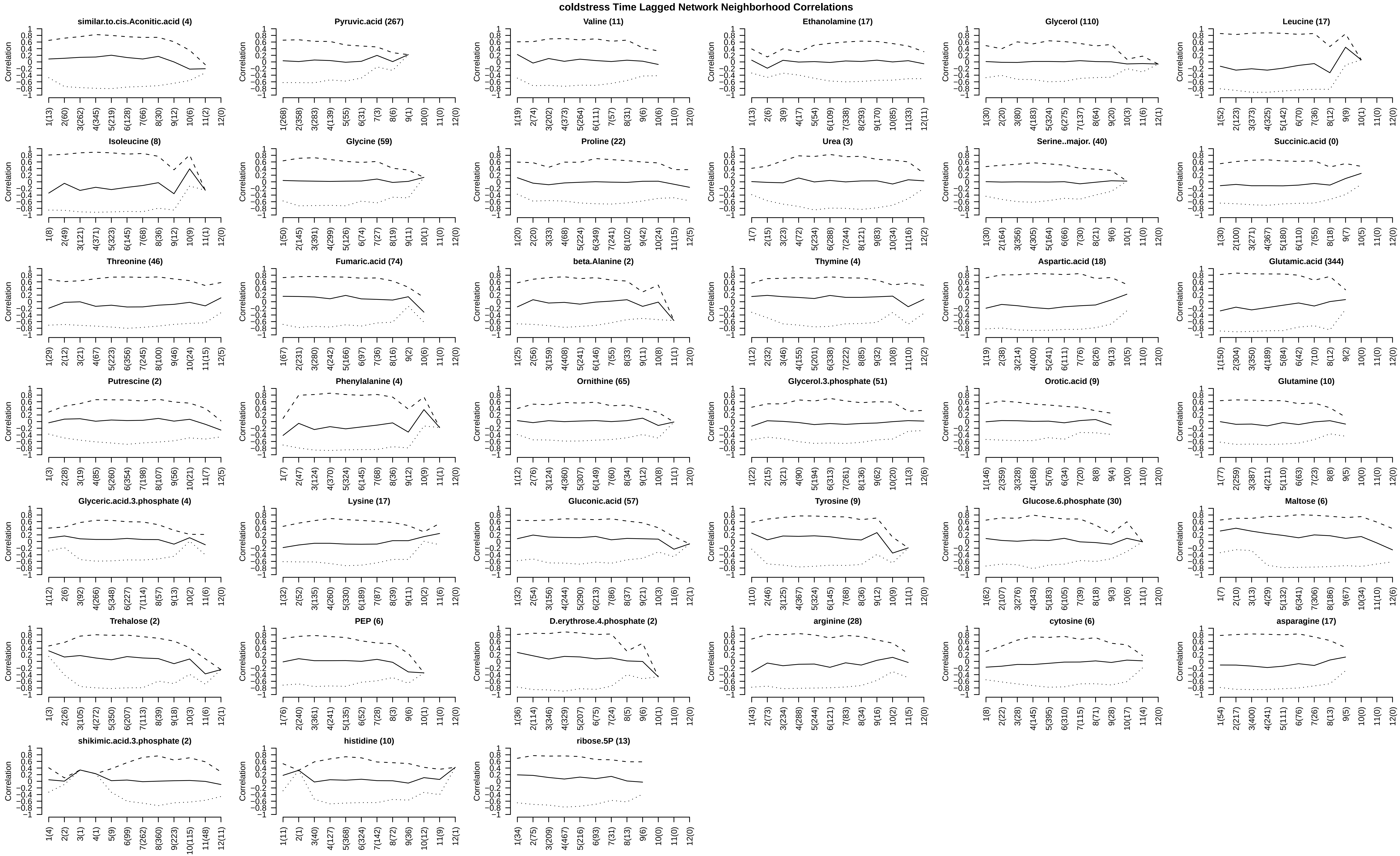
coldstress Time Lagged Network Neighborhood Correlations
similar.to.cis.Aconitic.acid (4)
Pyruvic.acid (267)
Valine (11)
Ethanolamine (17)
Glycerol (110)
Leucine (17)
Isoleucine (8)
Glycine (59)
Proline (22)
Urea (3)
Serine..major. (40)
Succinic.acid (0)
Threonine (46)
Fumaric.acid (74)
beta.Alanine (2)
Thymine (4)
Aspartic.acid (18)
Glutamic.acid (344)
Putrescine (2)
Phenylalanine (4)
Ornithine (65)
Glycerol.3.phosphate (51)
Orotic.acid (9)
Glutamine (10)
Glyceric.acid.3.phosphate (4)
Lysine (17)
Gluconic.acid (57)
Tyrosine (9)
Glucose.6.phosphate (30)
Maltose (6)
Trehalose (2)
PEP (6)
D.erythrose.4.phosphate (2)
arginine (28)
cytosine (6)
asparagine (17)
shikimic.acid.3.phosphate (2)
histidine (10)
ribose.5P (13)
Correlation

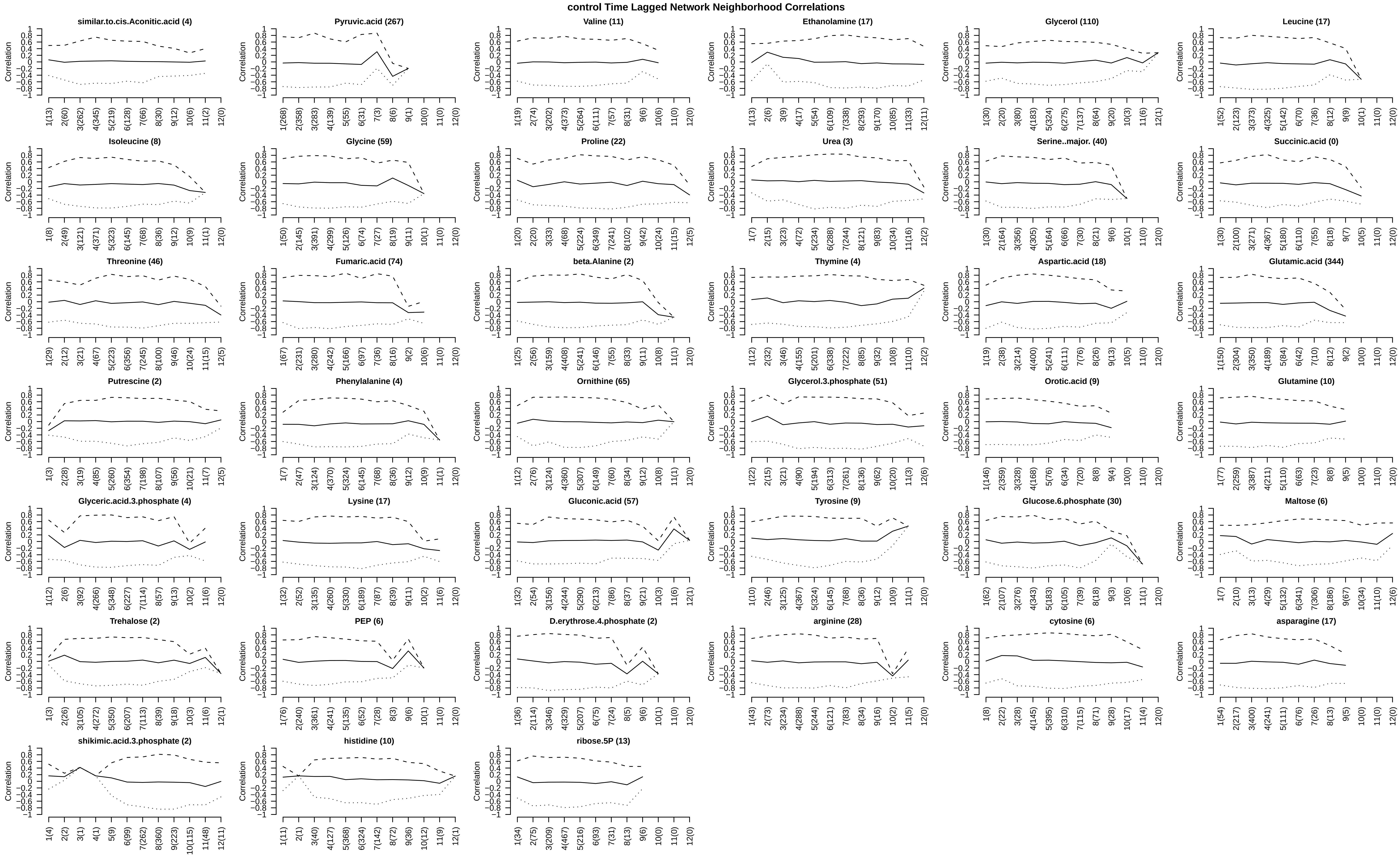
control Time Lagged Network Neighborhood Correlations
similar.to.cis.Aconitic.acid (4)
Pyruvic.acid (267)
Valine (11)
Ethanolamine (17)
Glycerol (110)
Leucine (17)
Isoleucine (8)
Glycine (59)
Proline (22)
Urea (3)
Serine..major. (40)
Succinic.acid (0)
Threonine (46)
Fumaric.acid (74)
beta.Alanine (2)
Thymine (4)
Aspartic.acid (18)
Glutamic.acid (344)
Putrescine (2)
Phenylalanine (4)
Ornithine (65)
Glycerol.3.phosphate (51)
Orotic.acid (9)
Glutamine (10)
Glyceric.acid.3.phosphate (4)
Lysine (17)
Gluconic.acid (57)
Tyrosine (9)
Glucose.6.phosphate (30)
Maltose (6)
Trehalose (2)
PEP (6)
D.erythrose.4.phosphate (2)
arginine (28)
cytosine (6)
asparagine (17)
shikimic.acid.3.phosphate (2)
histidine (10)
ribose.5P (13)
Correlation

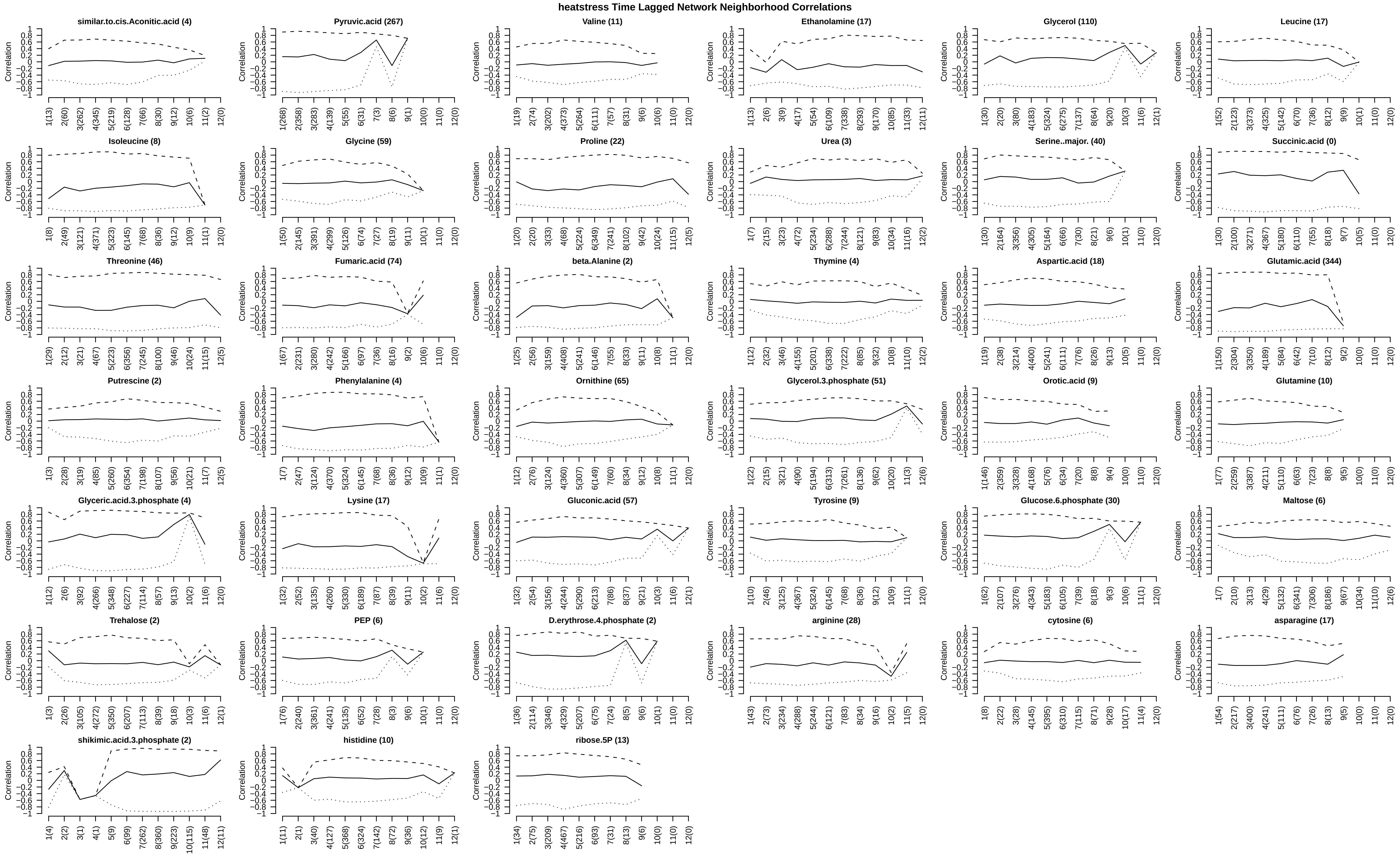
heatstress Time Lagged Network Neighborhood Correlations
similar.to.cis.Aconitic.acid (4)
Pyruvic.acid (267)
Valine (11)
Ethanolamine (17)
Glycerol (110)
Leucine (17)
Isoleucine (8)
Glycine (59)
Proline (22)
Urea (3)
Serine..major. (40)
Succinic.acid (0)
Threonine (46)
Fumaric.acid (74)
beta.Alanine (2)
Thymine (4)
Aspartic.acid (18)
Glutamic.acid (344)
Putrescine (2)
Phenylalanine (4)
Ornithine (65)
Glycerol.3.phosphate (51)
Orotic.acid (9)
Glutamine (10)
Glyceric.acid.3.phosphate (4)
Lysine (17)
Gluconic.acid (57)
Tyrosine (9)
Glucose.6.phosphate (30)
Maltose (6)
Trehalose (2)
PEP (6)
D.erythrose.4.phosphate (2)
arginine (28)
cytosine (6)
asparagine (17)
shikimic.acid.3.phosphate (2)
histidine (10)
ribose.5P (13)
Correlation

lactose Time Lagged Network Neighborhood Correlations

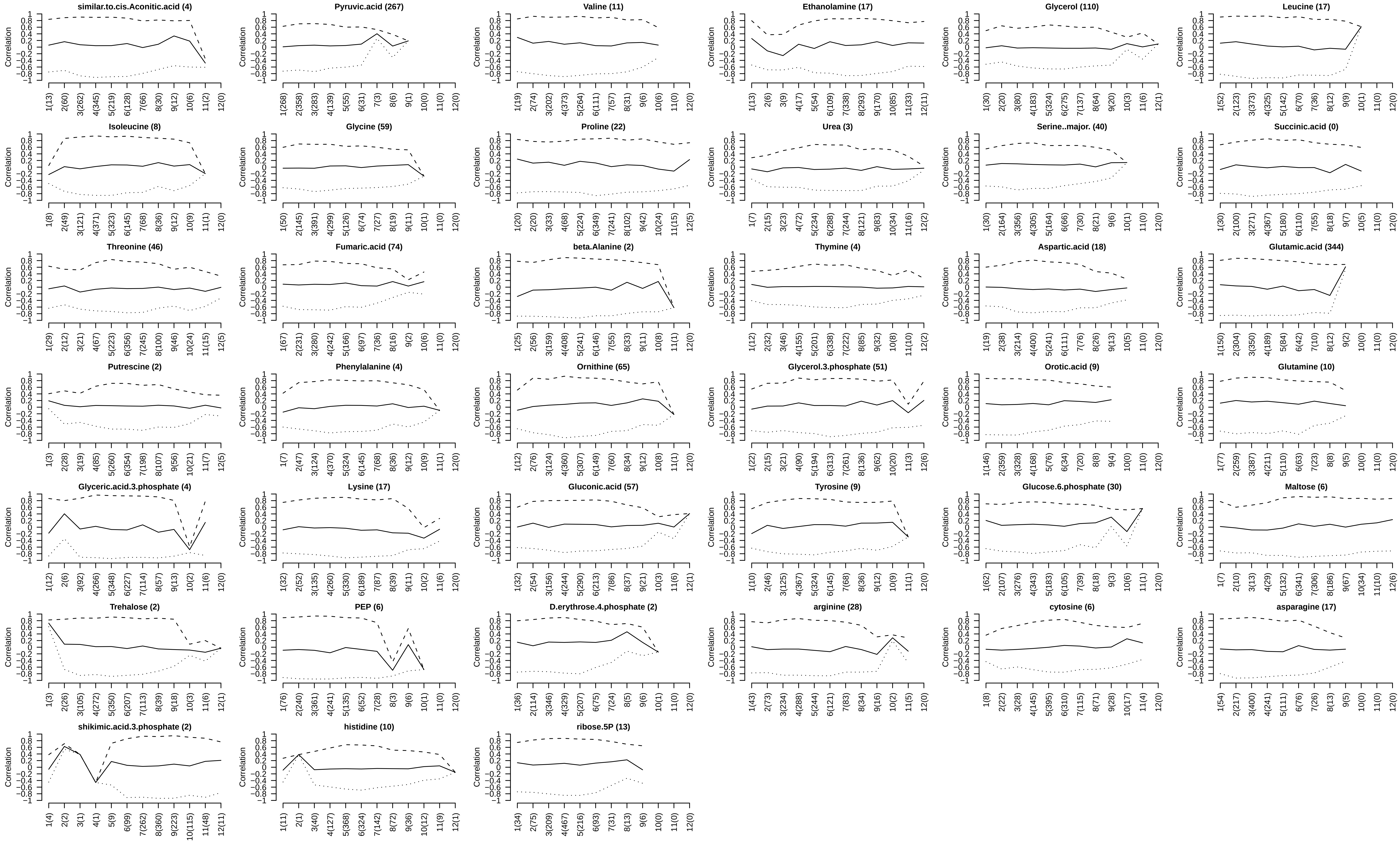

oxidativestress Time Lagged Network Neighborhood Correlations

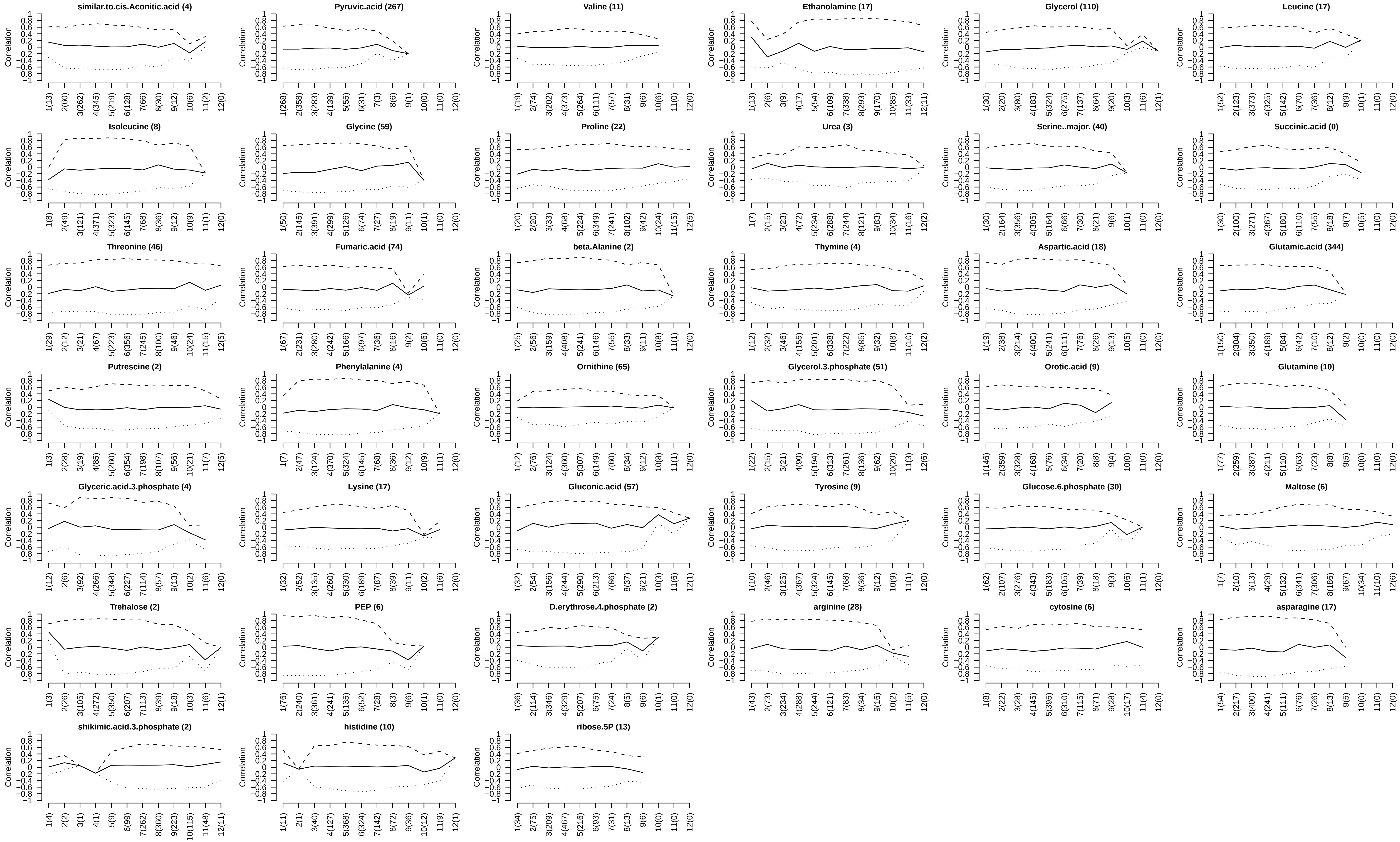

Supplement: Figure S1 — Maximum, mean and minimum correlation between metabolite concentration and gene expression at various network distances from each source metabolite. (PDF) [file pone.0031345.s001.pdf]
