# Supplementary figures and images for: Identifying Neighborhoods of Coordinated Gene Expression and Metabolite Profiles
Source: PLoS One. 2012 Feb 15;7(2):e31345. doi: 10.1371/journal.pone.0031345 (PMC3280297; doi:10.1371/journal.pone.0031345)

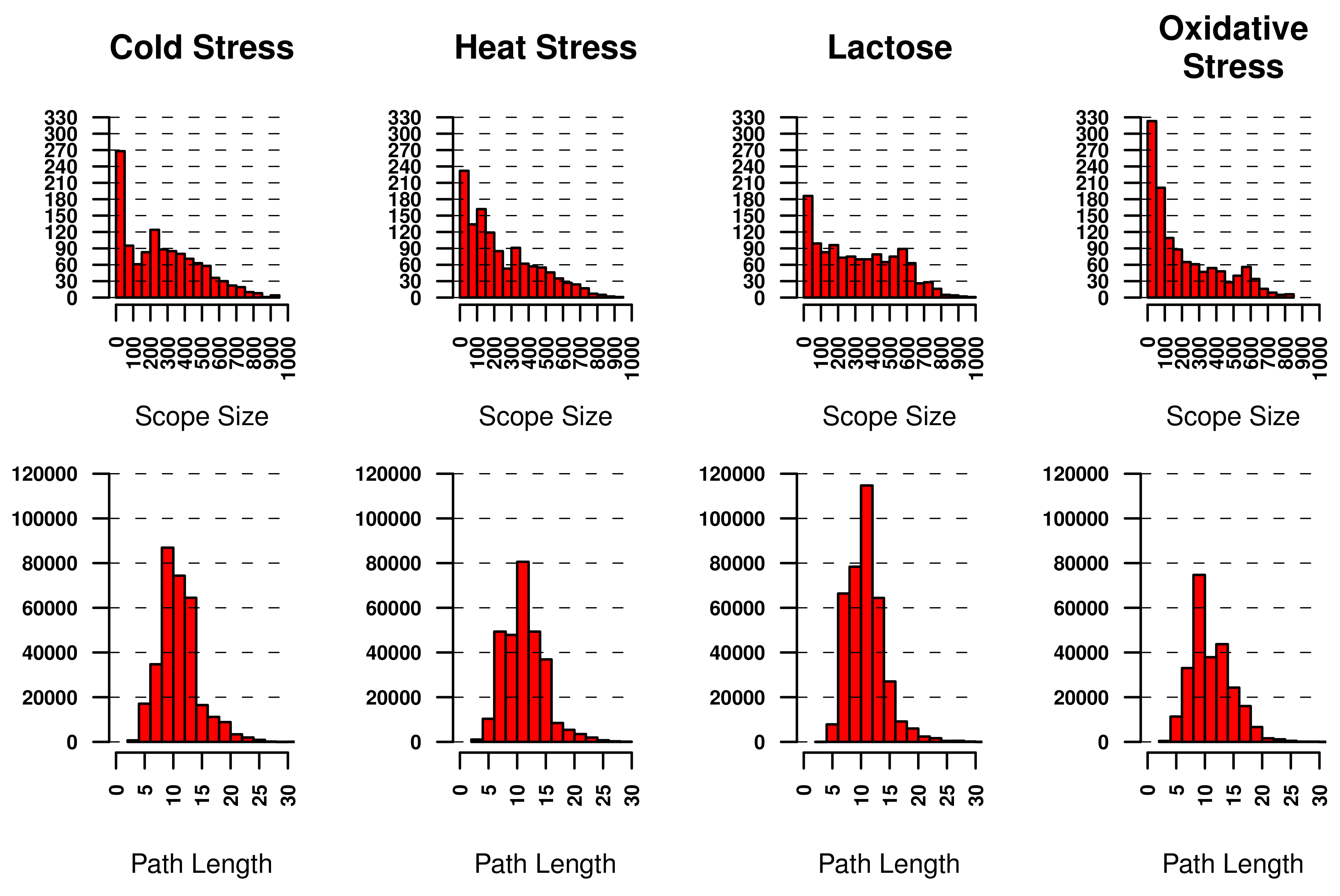

Supplement: Figure S2 — Scope size and path length distributions for each stress condition. (TIFF) [file pone.0031345.s002.tiff]
