# Supplementary material for: Identifying Neighborhoods of Coordinated Gene Expression and Metabolite Profiles
Source: PLoS One. 2012 Feb 15;7(2):e31345. doi: 10.1371/journal.pone.0031345 (PMC3280297; doi:10.1371/journal.pone.0031345)
Supplement: Figure S3 — Bipartite graph representing the structure of each stress conditions minimum set. Metabolite node sizes are proportional to the scope size of that metabolite. Hub reaction node size is proportional to how many times it is included in a minimum set metabolites scopes. In the case of long enzyme or metabolite names the first characters of the name are printed followed by the node label used within the SBML network file. The SBML file contains cellular compartment information for each metabolite {(c)ytosol, (e)xtraorganism, (p)eriplasm} which is printed after the ‘_’ in the metabolite name. (PDF) [file pone.0031345.s003.pdf]

1 Annotated Minimum Set Networks

We analyze the structure of the minimum sets by first extracting the most commonly traversed paths within the scope of each metabolite in the minimum set and then searching for common structure across all minimum set compound scopes. To extract the most commonly traversed paths within each metabolite's scope, we first collapse all extracted paths down into a single network. We then weight the edge of this network by the number of paths which include that network edge. Finally, we construct a maximum spanning tree to network the most commonly traversed paths. From these spanning trees we rank all nodes by their degree and extract the top ten nodes with the largest degree to define our hub reactions. From this list of minimum set metabolites and corresponding hub reactions we then construct a bipartite graph to summarize the structure of each minimum set. The bipartite graphs for each stress condition are shown in Supporting Figure S3.

In Supporting Figure S3 an edge between a minimum set metabolite and a hub reactions denotes the inclusion of that hub reaction within the scope of the minimum set compound. The relative sizes of the compound nodes indicate the size of each metabolite's scope and the size of the reaction node indicates the relative degree of each hub gene with the bipartite graph. Over all the stress conditions, the bipartite graphs show that the scopes within each minimum set are heavily overlapping as they share many common reaction hubs which form a large central sub-graph within the metabolic network. Although this large amount of overlap is expected as each minimum set includes over half the compounds and reactions within the entire network the sheer size of the central cluster and the number of common hubs illustrates the strong interconnectivity of metabolism. This interconnectivity reinforces the observation that metabolic networks are very robust as the density of these bipartite graphs is proportional to the number of paths which connect the critical metabolites.

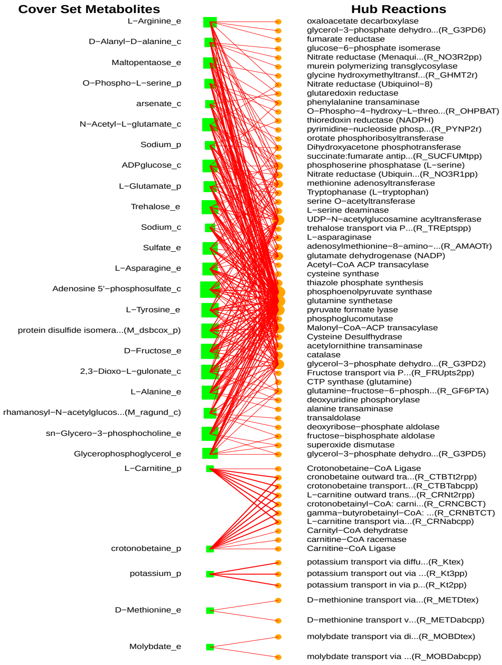

(a) Cold stress.

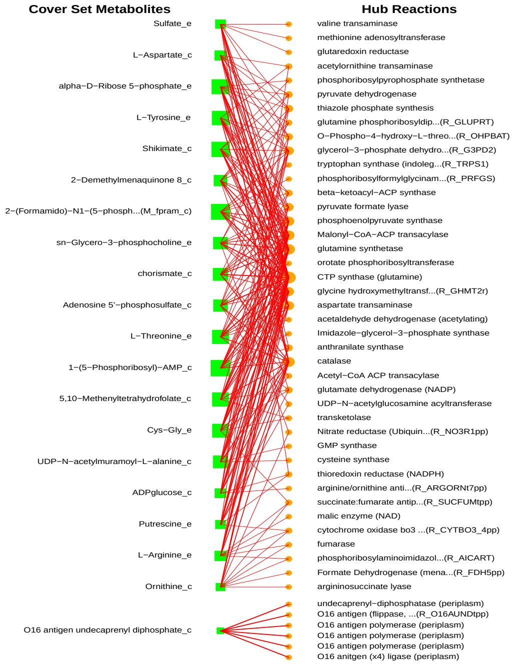

(b) Heat stress.

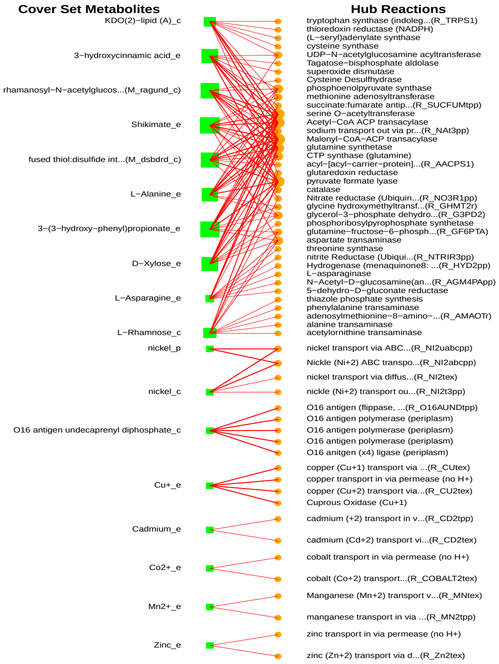

(c) Lactose stress

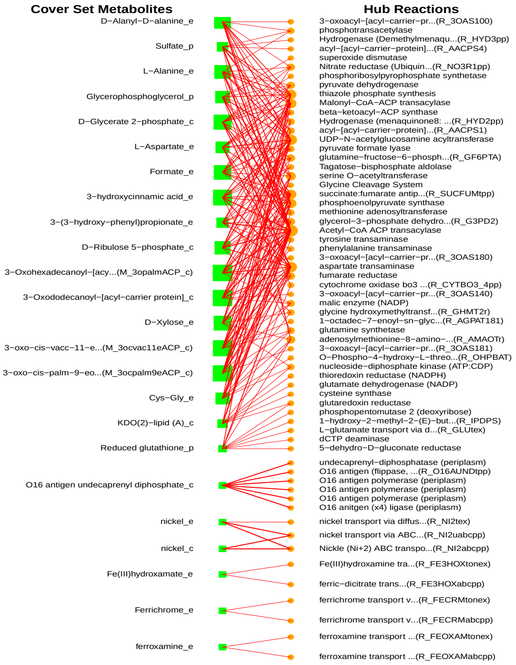

(d) Oxidative stress

Supporting Figure S3: Bipartite graph representing the structure of each stress conditions minimum set. Metabolite node sizes are proportional to the scope size of that metabolite. Hub reaction node size is proportional to how many times it is included in a minimum set metabolites scopes. In the case of long enzyme or metabolite names the first characters of the name are printed followed by the node label used within the SBML network file. The SBML file contains cellular compartment information for each metabolite {(c)ytosol, (e)xtraorganism, (p)eriplasm} which is printed after the '_' in the metabolite name.
