# Supplementary material for: Identifying Neighborhoods of Coordinated Gene Expression and Metabolite Profiles
Source: PLoS One. 2012 Feb 15;7(2):e31345. doi: 10.1371/journal.pone.0031345 (PMC3280297; doi:10.1371/journal.pone.0031345)
Supplement: Methods S1 — (PDF) [file pone.0031345.s004.pdf]

Supporting Methods: Identifying Neighborhoods of Coordinated Gene Expression and Metabolite Profiles

Timothy Hancock[1*], Nicolas Wicker[2], Ichigaku Takigawa[1], Hiroshi Mamitsuka[1]
1 Bioinformatics Center, Kyoto University, Japan.
2 Laboratoire de Bioinformatique et Génomique Intégratives, Institut de Génétique et de Biologie Moléculaire et Cellulaire, Université de Strasbourg, France.
∗ E-mail: timhancock@kuicr.kyoto-u.ac.jp

Network Processing

The specific network reconstruction used is iAF1260 for *Escherichia coli K-12 MG1655* [Feist *et al* (2007)] and was sourced from the BiGG database [Schellenberger *et al* (2010)]. This network contains 1972 unique metabolic compound entries and 1944 reaction entries. Before any pathway analysis the network was preprocessed into a network of connected reactions. The preprocessing connects neighboring reactions by their substrate and product compound dependencies. In Diagram 1a we present an example compound-reaction network pathway as is presented in many metabolic databases. The example network in Diagram 1a connects six compounds $\{c_1, \ldots, c_6\}$ by two reactions $\{R_1, R_2\}$ through the substrate-product compound dependency of c_3. The converted reaction network of Diagram 1a is presented in Diagram 1b. For ease of the pathway interpretation we have included additional pseudo-nodes $\mathbf{s}$ and $\mathbf{t}$ which flag the entrance substrates $\mathbf{s} = \{c_1, c_2\}$ and the terminal products $\mathbf{t} = \{c_4, c_5, c_6\}$ of the network. In Diagram 1b the edges are clearly labeled by a tuple containing the initial substrate, intermediate product and resulting product compounds traversed by each path through the two reactions. By following the edge labels in Diagram 1b every possible compound transition path within Diagram 1a can be recovered. Additionally as each path is uniquely labeled by the compound transitions the direction of the edge can be maintained. Edge weights w are then assigned between each connected reaction pair to be the maximum Pearson correlation between the expression profiles computed for all pairwise gene combinations from the gene sets of each connected reaction gene set space.

Preprocessing in this manner treats each substrate-product pair as equally important and the edge labeled by the specific substrate-product pairs are used to ensure that information on the specific location of each pair within the network and edge direction between them is maintained. However, treating each substrate-product pair as a separate path does violate the requirement that all substrates must be present before the reaction can proceed. However, as the edge weights that connect the same reactions are identical, each substrate-product pair resulting from each connected reaction is equally likely. Therefore, as the specific reactions which are selected to be a member of a maximally correlated path will also depend on the coordination of the neighboring reactions further down the path, it is assumed that specific reactions selected within each path will be indicative of the major compound transformations along the most biologically meaningful path.

Significant Path Ranking

To compute path significance we first define the score of each path π to be score s_π (1),

$$s_\pi = \prod_{k=1}^{|\pi|-1} P_{ECDF}\left(w \le w_{R_k \to R_{k+1}}\right) \tag{1}$$

where $|\pi|$ is the path length and P_{ECDF} is the empirical cumulative distribution probability of an edge weight $w_{R_k \to R_{k+1}}$ given all other edge weights within the network. If we then assume that the edges

along a given path are randomly and independently drawn from the network edge weight distribution, the p-value of the path can be computed using (2) [Takigawa and Mamitsuka (2008)].

$$P(Y \geq s) = 1 - s \sum_{i=0}^{|\pi|-1} \frac{(-ln(s))^i}{i!} \tag{2}$$

From [Takigawa and Mamitsuka (2008),Hancock *et al* (2010)] we know that ranking only by (1) is biased towards shorter path lengths. However ranking by p-value corrects the path length dependency but induces an assumption that each edge weight is randomly and independently drawn from the network edge weight distribution, which given the known network structure is unlikely to hold. In our proposed pathway ranking algorithm we address both of these concerns with a combination of a dynamic programming pathway extraction algorithm to extract the path of smallest p-value and a Metropolis sampling regime to correct underlying assumptions of this p-value computation.

From observation of the p-value computation in (2) we see that if we hold the path length constant, the task of finding the path of minimum p-value is equivalent to maximizing the path score function (1). This suggests an algorithm that computing shortest paths in terms of score for all lengths and then ranking by the p-value of each path would yield an algorithm to find the path of minimum p-value. To compute the shortest paths (in number of edges) we propose a dynamic programming to efficiently account for path length.

In Diagram 2 we show a diagrammatic representation of the algorithm for extracting all shortest paths, in terms of score (1), for all lengths 0 to $|\pi|^{\max} - 1$ between $\mathbf{s}$ and $\mathbf{t}$. Diagram 2 shows that if we have a list of all shortest paths, in terms of score for all lengths, to all nodes directly connected to $\mathbf{t}$, $[R_0 \ldots R_k]$, we can readily find the shortest path to $\mathbf{t}$ at all lengths by selecting the edge connected to $\mathbf{t}$ with the minimum weight $s_{s \to R_k} + w_{R_k \to t}$. This logic can be followed recursively through the network to find the shortest path of all lengths to all nodes and results in the dynamic programming algorithm presented in Diagram 3. The algorithm in Diagram 3 defines the weights of edges $R_k \to R_{k+1}$ as $w_{R_k \to R_{k+1}} = -\log_2(w_{R_x,R_y})$, and if edge $R_k \to R_{k+1}$ does not exist, then $w_{R_k \to R_{k+1}} = +\infty$. Once the shortest path in terms of score for all lengths has been found, the path with smallest p-value can be easily recovered by ranking all shortest paths with respect to (2).

To correct the p-value computation given that the path edge weights are unlikely to be randomly and independently drawn from the network edge weight distribution, we employ a Metropolis sampling algorithm [Metropolis *et al* (1953)]. The resulting Metropolis algorithm (Diagram 4) randomly samples candidate paths π^* of all lengths 0 to $|\pi|$ from the weighted network. The probability $p(\pi^*)$, of each randomly sampled path π is stored and used as a reference distribution to compute the p-value of each shortest path identified by the algorithm in Diagram 3. We now show that computing the p-value from this reference distribution overcomes the assumption that path edge is randomly and independently drawn from the network distribution.

For each path generation step within Diagram 4, using the Metropolis algorithm [Metropolis *et al* (1953)], each vertex transition is accepted with probability $1 \wedge \frac{pr(\pi^*)p(x)}{pr(x)p(\pi^*)}$ where by pr and p are the target and proposal distribution respectively. As the target distribution is the uniform distribution, this simplifies to $1 \wedge \frac{p(x)}{p(\pi^*)}$. However, this expression is still not easily evaluated, as indeed algorithm 4 can fail, and the probability $p(\pi)$ is not simply equal to a random choice of edges given the degree distribution $g(\pi) = \left(\prod_{i \in 1,\ldots,l} d(x_i)\right)^{-1}$ where $d(x_i)$ is the degree of the ith vertex of path π, but is dependent on the network structure. We now want wish to assess if such a random sampling procedure as described in Diagram 3 will faithfully reconstruct the distribution of all paths through the network.

Over the course of many trials $t \in [1, \ldots, T_{\max}]$ the convergence of this generated random distribution, $g(\pi)$, to the target distribution of paths through the network $p(\pi)$ can be assessed by considering the error between the $g(\pi)$ and $p(\pi)$ when going through one path generation step and arriving at a cycle or at a dead-end. In the cycle case the process arrives at a vertex which has already been chosen, and a

dead-end path occurs when there are no more edges to choose while the objective length has not yet been reached. In both of these cases no path is produced by our path sampling or shortest path algorithms. Let us denote h to be the probability of producing no path. We can now evaluate the probability $p(\pi)$ of observing the path π. If one observes π, this can be at the first trial, then it is produced with probability $g(\pi)$, and after t trials and then the probability becomes $g(\pi)h^{t-1}$ and therefore becomes a geometric progression and can be shown to converge to the network path distribution (3).

$$p(\pi) = \sum_{t=0}^{+\infty} g(\pi)h^t = \frac{g(\pi)}{1-h} \text{ as } h < 1$$

Initially, h is set to 0 as $p(\pi)$ is considered as being equal to $g(\pi)$, and then at each sampling trial h is corrected using the proportion of observed errors and increasingly becomes more reflective of the known network structure. This ensures the convergence of the algorithm and shows that as the number of trials increases our sampled path probability distribution will resemble that of the network.

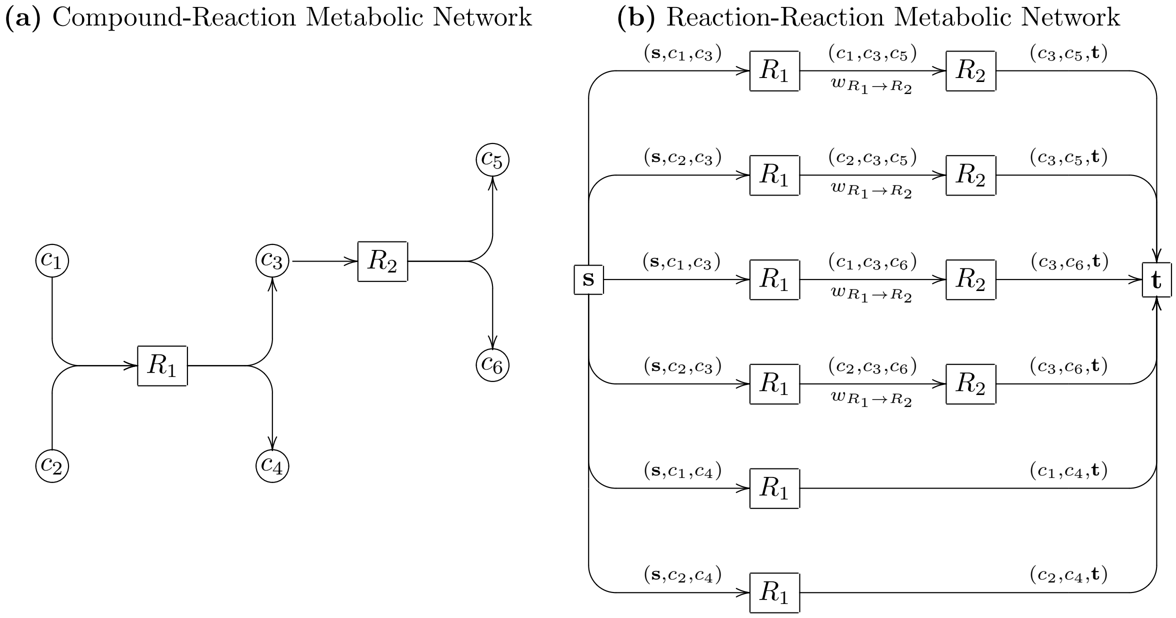

Supporting Methods Diagram 1. Diagrammatic example of a compound-reaction metabolic network into a reaction-reaction metabolic network. In this example there are six metabolic compound $\{c_1, \ldots, c_6\}$ and two reactions $\{R_1, R_2\}$ connected by a substrate-product dependency through compound c_3. Edge weights are labeled as w.

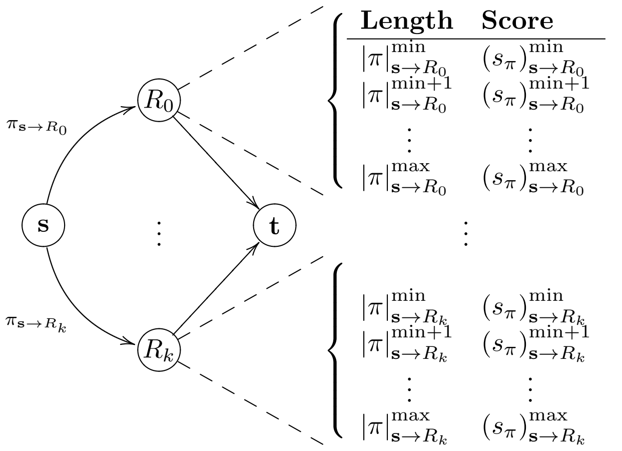

Supporting Methods Diagram 2. Diagrammatic representation of how to choose the shortest path.

$\forall i \in 1, \ldots, n$ and $\forall R_k$ $(s_\pi)^i_{\mathbf{s}\to R_k} = +\infty$ and $(s_\pi)^0_{\mathbf{s}\to\mathbf{s}} = 0$
for l to $|\pi| - 1$ **do**
  for all R_k s.t. $(s_\pi)^l_{\mathbf{s}\to R_k} \neq +\infty$ **do**
    for all R_{k+1} s.t. $w_{R_k\to R_{k+1}} < +\infty$ **do**
      if $(s_\pi)^l_{\mathbf{s}\to R_k} + w_{R_k\to R_{k+1}} < (s_\pi)^{l+1}_{\mathbf{s}\to R_k}$ **then**
        $(s_\pi)^{l+1}_{\mathbf{s}\to x} \leftarrow (s_\pi)^l_{\mathbf{s}\to R_k} + w_{R_k\to R_{k+1}}$
      end if
    end for
  end for
end for

Supporting Methods Diagram 3. Algorithm for finding the shortest path between **s** and **t**.

---

for t in $T_{\max}$ **do**
  randomly choose a starting vertex from the network **s**.
  $v \leftarrow s$ and $\pi = \mathbf{s}$
  while $|\pi| < l + 1$ **do**
    choose uniformly randomly a neighbor vertex u of v which does not make a cycle
    if v not found **then**
      return ERROR
    else
      $\pi \leftarrow \pi.u$ and $v \leftarrow u$
    end if
  end while
  store π
end for

Supporting Methods Diagram 4. Pathway sampling distribution construction.

References

Feist *et al* (2007). Feist AM, Henry CS, Reed JL, Krummenacker M, Joyce AR, Karp PD, Broadbelt LJ, Hatzimanikatis V, Palsson BØ (2007) A genome-scale metabolic reconstruction for Escherichia coli K-12 MG1655 that accounts for 1260 ORFs and thermodynamic information. *Mol Syst Biol* **3**: 121

Hancock *et al* (2010). Hancock T, Takigawa I, Mamitsuka H (2010) Mining metabolic pathways through gene expression. *Bioinformatics* **26**: 2128–2135

Matthews *et al* (2009). Matthews L, Gopinath G, Gillespie M, Caudy M, Croft D, de Bono B, Garapati P, Hemish J, Hermjakob H, Jassal B, Kanapin A, Lewis S, Mahajan S, May B, Schmidt E, Vastrik I, Wu G, Birney E, Stein L, D'Eustachio P (2009) Reactome knowledgebase of human biological pathways and processes. *Nucleic Acids Res* **37**: D619–22

Metropolis *et al* (1953). Metropolis N, Rosenbluth AW, Rosenbluth MN, Teller AH, Teller E (1953) Equations of state calculations by fast computing machines. *Journal of Chemical Physics* **21 or 6**: 1087–1091

Schellenberger *et al* (2010). Schellenberger J, Park JO, Conrad TM, Palsson BØ (2010) BiGG: a Biochemical Genetic and Genomic knowledgebase of large scale metabolic reconstructions. *BMC Bioinformatics* **11**: 213

Takigawa and Mamitsuka (2008). Takigawa I, Mamitsuka H (2008) Probabilistic path ranking based on adjacent pairwise coexpression for metabolic transcripts analysis. *Bioinformatics* **24**: 250–257

Vastrik *et al* (2007). Vastrik I, D'Eustachio P, Schmidt E, Gopinath G, Croft D, de Bono B, Gillespie M, Jassal B, Lewis S, Matthews L, Wu G, Birney E, Stein L (2007) Reactome: a knowledge base of biologic pathways and processes. *Genome Biol* **8**: R39
